# A rare cause of mechanical bowel obstruction: ingested vinyl glove. Unexpected diagnosis at laparoscopy

**DOI:** 10.1002/ccr3.796

**Published:** 2017-04-04

**Authors:** Nazareno Smerieri, Italo Barbieri, Matteo Fumagalli, Davide Luppi, Andrea Lanaia, Stefano Bonilauri

**Affiliations:** ^1^ General and Emergency Surgery UnitAzienda Ospedaliera Arcispedale Santa Maria Nuova ‐ IRCCSViale Umberto I50, Reggio Emilia42123Italy

**Keywords:** Acute abdomen, foreign body, laparoscopy, small bowel obstruction, vinyl glove

## Abstract

The report suggests that, when the patient's history, clinical examination, and findings do not lead to a clear diagnosis in case of an acute abdomen, a laparoscopic approach, that has both, diagnostic and therapeutic value, is advised.

A 15‐year‐old girl, with a negative past medical history, was referred to our department with right lower quadrant pain, tenderness, and rebound pain. She presented with nausea and vomiting. She had no fever but complained of constipation. Blood tests demonstrated leukocytosis and an elevated C‐reactive protein. An abdominal X‐Ray showed centrally located bowel distension, with presence of gas‐fluid levels (Fig. 1). Ultrasonography identified only a localized pelvic fluid collection. A peritonitis due to acute appendicitis, with concomitant dynamic ileus, was suspected, and a small bowel obstruction as an alternative. During laparoscopy, pelvic fluid collection was confirmed; there was no evidence of acute appendicitis, but a distinct transition zone between normal and abnormally dilated small bowel was found, caused by a solid material in the intestinal lumen (Fig. 2).

**Figure 1:**
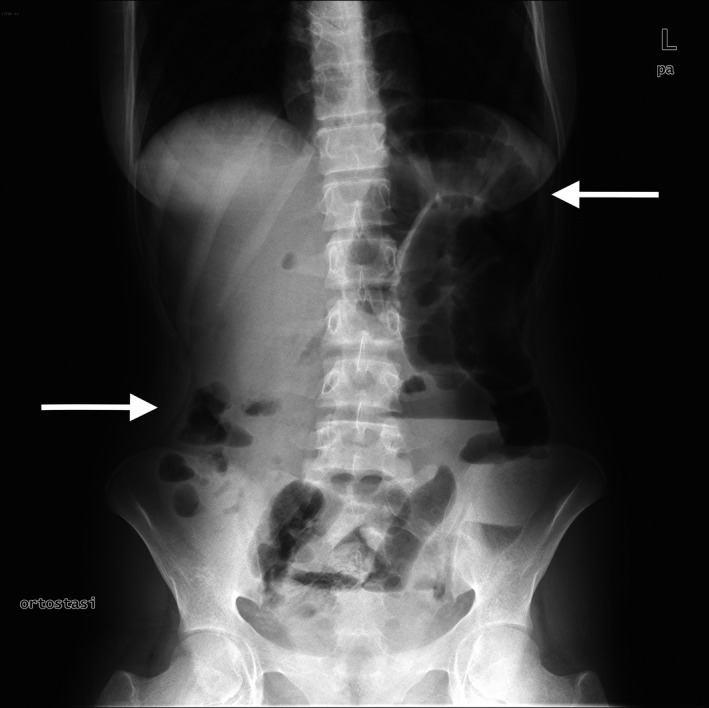
X‐Ray shows centrally located bowel distension with presence of gas‐fluid levels.

**Figure 2:**
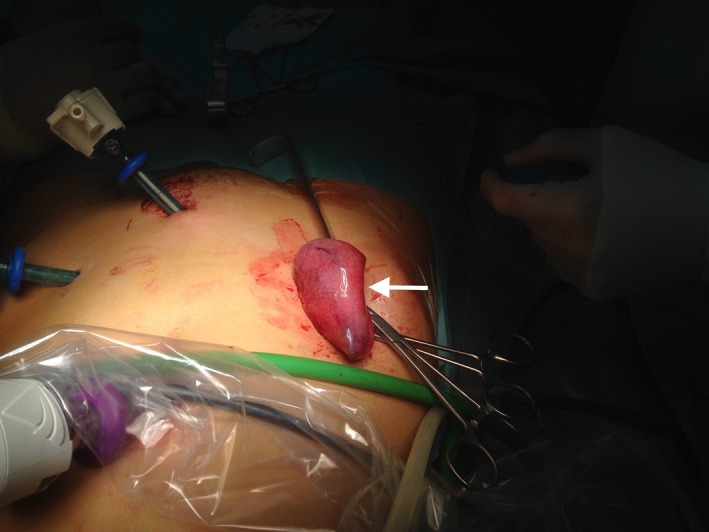
The abnormally dilated small bowel is exteriorized.

## What is this?

**Figure 3:**
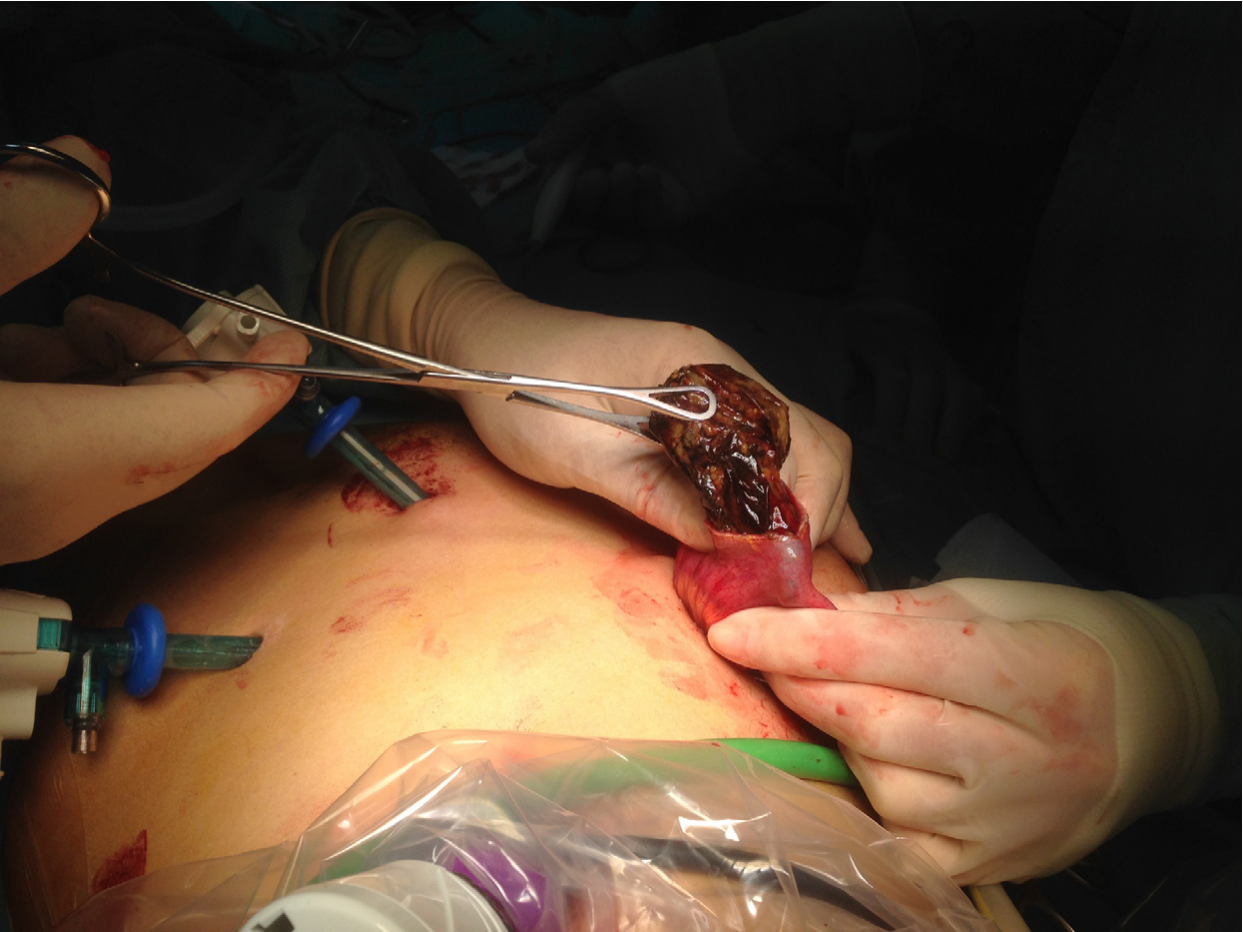
Enterotomy shows an intraluminal foreign body.

**Figure 4:**
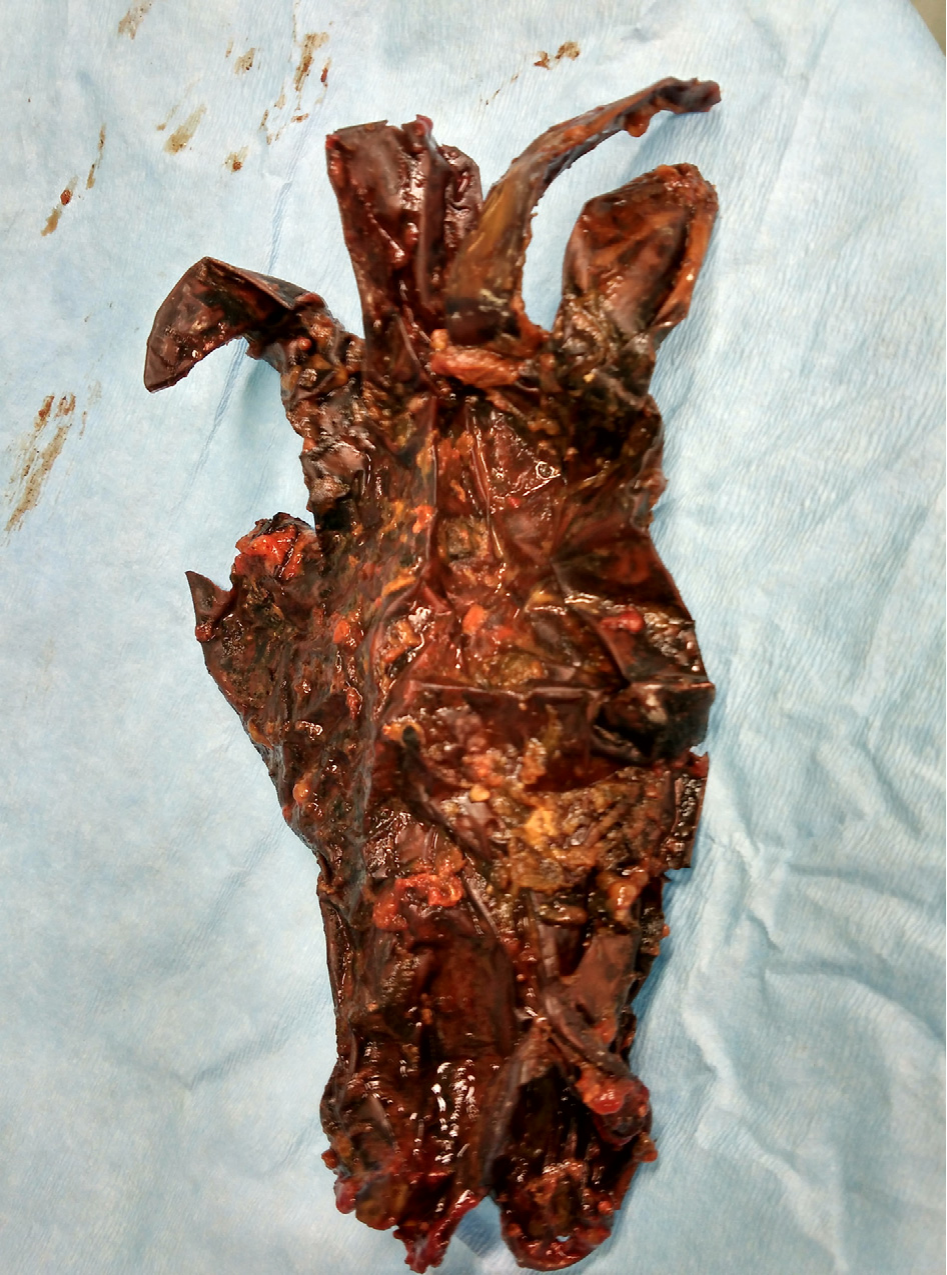
The vinyl glove.

Enterotomy revealed the intraluminal presence of a vinyl glove causing small bowel obstruction (Figs 3, 4). After the procedure, both the patient and her parents denied any previous strange behavior or glove ingestion. She was discharged 3 days post‐op and referred to neuropsychiatric unit.

Foreign body ingestion, by accidental means, is most commonly seen in the pediatric population, and the management is still controversial 1. However, intentional ingestion of foreign bodies has been reported in adults with mental health issues, in prisoners, or body packers 2, 3. We report a rare case of small bowel obstruction due to vinyl glove ingestion, in order to highlight the challenge of differential diagnosis in such case. When ingested, vinyl gloves undergo chemical transformation, likely a result of cross‐bridging of polymers, similar to the process of rubber vulcanization. This process could result in small bowel obstruction or perforation 4, 5.

## Authorship

NS: participated in study conception and design. NS, IB, MF, DL, AL and SB: performed acquisition of data. NS, IB: involved in analysis and interpretation of data. NS: drafted the manuscript. NS, IB, MF, DL, AL and SB: made critical revision. NS: performed operation.

## Conflict of Interest

None declared.
